# Pyrometamorphic process of ceramic composite materials in pottery production in the Bronze/Iron Age of the Northern Caucasus (Russia)

**DOI:** 10.1038/s41598-019-47228-y

**Published:** 2019-07-24

**Authors:** Ki Suk Park, Ralf Milke, Ilias Efthimiopoulos, Regine-Ricarda Pausewein, Sabine Reinhold

**Affiliations:** 1 Institut für Geologische Wissenschaften, Freie Universität Berlin, Malteserstraße 74-100, 12249 Berlin, Germany; 2 Deutsches GeoForschungsZentrum GFZ, Section 3.6, Telegrafenberg, 14473 Potsdam Germany; 3 RathgenForschungslabor, Staatliche Museen zu Berlin, Schloßstraße 1, 14059 Berlin, Germany; 4 Deutsches Archäologisches Institut, Im Dol 2-6, Haus II, 14195 Berlin, Germany

**Keywords:** Mineralogy, Sedimentology, Environmental social sciences

## Abstract

Pyrotechnology for the prehistoric pottery has been an important subject for the study of ancient production technology and technological styles. However, heterogeneous characteristics in chemical and mineralogical compositions and massive amounts of ceramic sherds at most archaeological sites make it difficult to identify production technologies. In this study, SEM-EDS/WDS, XRD and transmittance and reflectance FT-IR techniques were employed step by step, in order to overcome these limitations. The serial combination of each method covers a macro-, meso- and micro-scale and it enabled us to identify the relationship between firing temperature, reducing or oxidizing atmosphere and thermally induced mobility of Ca and Fe. Numerous ceramic pottery sherds from two archaeological sites in the North Caucasus, Ransyrt 1 (Middle-Late Bronze Age) and Kabardinka 2 (Late Bronze/Early Iron Age) were investigated and compared to the ceramics found at Levinsadovka and Saf’janovo around the Sea of Azov, Russia (Late/Final Bronze Age) for this purpose. Morphological changes by sintering and transformation of indicator minerals such as calcite, hematite, spinel, gehlenite, quartz and *cis/trans-vacant* 1M illite provide temperature thresholds at 675, 700, 750, 950, 1050, 1100, 1300 °C. With the laboratory based FT-IR, vibrational changes in shape, wavenumber and intensity corresponding to Si-O stretching bands yield an order and classification of the ceramics with regard to firing conditions between the samples as well as the unraveling of temperature profiles within a single sample in a 100 µm scale. With this approach, the number of archaeological ceramics could be classified according to the pyrometamorphic transformation of heterogeneous ceramic composite materials. Combined with the archaeological contexts of each site, these results will contribute to the reconstruction of local technological styles.

## Introduction

Pyrotechnology in the prehistoric society has been an important topic in the archaeological ceramic studies. Because the control of fire was a critical issue for the prehistoric potters, identification of firing technology is necessary to understand various technological variation in local societies. In the prehistoric craft system, this firing technology was transferred through practice in small-scale interpersonal relations^1^ and characteristic ways of controlling fire in various conditions designated local technological styles^2–4^. However, heterogeneity in the mineralogical and chemical composition and thermal property of most prehistoric ceramics make it difficult to identify production technologies and technological styles. For example, illite occurs in the prehistoric ceramics as one of the most common clay minerals. However, its polytypes have heterogeneous thermal decomposition procedure and were dehydroxylated in different temperatures under heating^5,6^. Various sized sand grains composed of different minerals in the ceramics will interrupt the precise interpretation of the firing state. Furthermore, most archaeological sites yield numerous ceramic sherds which carry different technological styles within a same site. Previous researches studying production technologies of archaeological ceramics have focused either only on the chemical composition in a macro-scale or firing behaviors of a few specific mineral phases. These approaches are still difficult to answer the question about the various production techniques imprinted in a large number of heterogenous ceramics. In this study, various analytical instruments such as SEM-EDS/WDS (Scanning Electron Microscopy with Energy/Wavelength-Dispersive X-ray Spectroscopy), XRD (X-ray Powder Diffraction) and FT-IR (Fourier-Transform Infrared Spectroscopy, transmittance and reflectance) are employed step by step, in order to solve these problems. Samples will be measured first for a macro-scale and categorized into data groups. From these groups, representative samples will be selected for the further measurement with a higher resolution. With this approach, the data representing various firing conditions can be gathered efficiently from numerous samples. Moreover, average firing conditions estimated from macro-scale observations as well as more precise pyrometamorphic state within a sample from a meso-/micro-scale can be compared to each other, so that the more precise categorization and interpretation are possible.

In this study, this serial combination of the various methods and measurement scales will be employed for the daily ware ceramics excavated in the high plateaus of the North Caucasus in the Bronze Age and the Iron Age. It is expected that firing temperature ranges and atmospheric conditions of heterogeneous archaeological ceramics can be identified for the reconstruction of local technological styles in the daily ware production.

## Archaeological Sites

**Figure 1:**
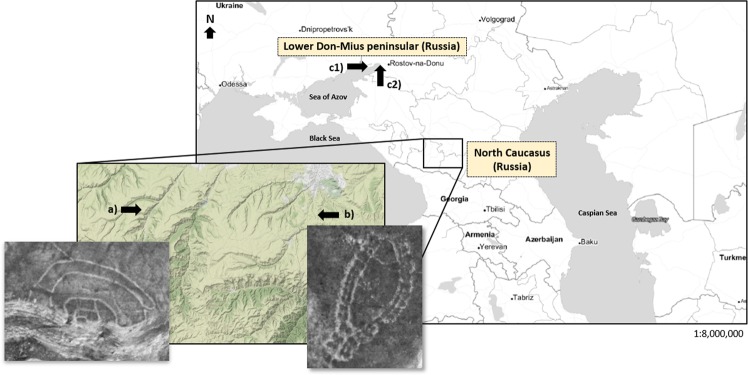
Archaeological sites in North Caucasus and in the northern Black Sea coast: a) Ransyrt 1; b) Kabardinka 2; c1) Levinsadovka (Mius peninsular); c2) Saf’janovo (Lower Don) (maps: created by QGIS 2.18.0 with open layers from OSM/Stamen, map tiles by Stamen Design, under CC BY 3.0. data by OpenStreetMap, under ODbL (maps.stamen.com); photos: Reinhold *et al*.^7^).

Figure 1 describes the archaeological sites of this study. Ransyrt 1 is located on the plateau with the height of 1850 m above sea level in Karachay-Cherkess Republic of the Russian Federation (43°50′29.7″N, 42°18′10.3″E). Kabardinka 2 lies on the lower plateau with 1400 m a.s.l. in Stavropol Krai of the Russian Federation (43°49′40.9″N, 42°42′57.4″E). The objects were excavated by the joint project of German Archaeological Institute (S. Reinhold), the Institute of Archaeology, Russian Academy of Sciences (D.S. Korobov) and GUP Nasledie, heritage organization in Stavropol, Russia (A.B. Belinsky) between 2006–2008 and 2013–2015. According to the local chronology defined by the construction phases and ^14^C data, Ransyrt 1 is dated to 1800–1500 BC, the Middle Bronze Age (MBA) to the Late Bronze Age (LBA) and Kabardinka 2 to 1600–800 BC, which belong to the LBA and Early Iron Age (EIA)^5^. Especially Kabardinka 2 has relatively longer occupation history proved by two different construction phases, i.e., a linear phase between 1600–1200 BC and a symmetric phase between 1300–800 BC. In this time period, Koban culture was known in the North Caucasus region^7^. Geologically, Ransyrt 1 bedrock is composed of dolomite, while the bedrock of Kabardinka 2 is mainly composed of calcite. Soil development of Kabardinka 2 is more progressed than Ransyrt 1.

These mountain ceramics were compared to the other archaeological ceramics from Levinsadovka (47°10′9.9″N, 38°30.17″E) and Saf’janovo (47°15′59.7″N, 39°26′30.1″E), located on the coast of Mius peninsular and on the lower area of the Don river. They were excavated by another joint project of the German Archaeological Institute, Don-Archaeological Society (Rostov on Don) and Institute of Archaeology, Russian Academy of Sciences. The site at Levinsadovka on the Mius peninsular was occupied by the Late Srubnaja Culture (LBA) and that at Saf’janovo by Kobjakovo Culture (Final Bronze Age, FBA)^8^. Corresponding to the radio carbon data, the both cultures were overlapping between 1600–800 BC.

## Results

### Mineralogical composition of sand and silt grains in the ceramics

**Figure 2:**
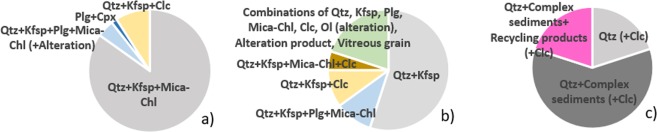
Dominant mineralogical combinations of the ceramics from (**a**) Ransyrt 1, (**b**) Kabardinka 2, 3) Levinsadovka-Saf’janovo (Clc: calcite, Cpx: clinopyroxene, Kfsp: K-feldspar, Mica-Chl: mica-chlorite mixed layers, Ol: olivine, Plg: plagioclase, Qtz: quartz).

Most sand and silt grains in the ceramics are lithoclasts derived from volcanic, metamorphic or sedimentary rocks. Various mineralogical combinations in the ceramic pastes besides clay minerals were identified by optical petrography, XRD and SEM-EDS and it is summarized according to the archaeological site (Fig. 2). The chemical composition of all the alteration products or the products of mineral intergrowth were measured by SEM-WDS (see Supplementary Table S1).

**Figure 3:**
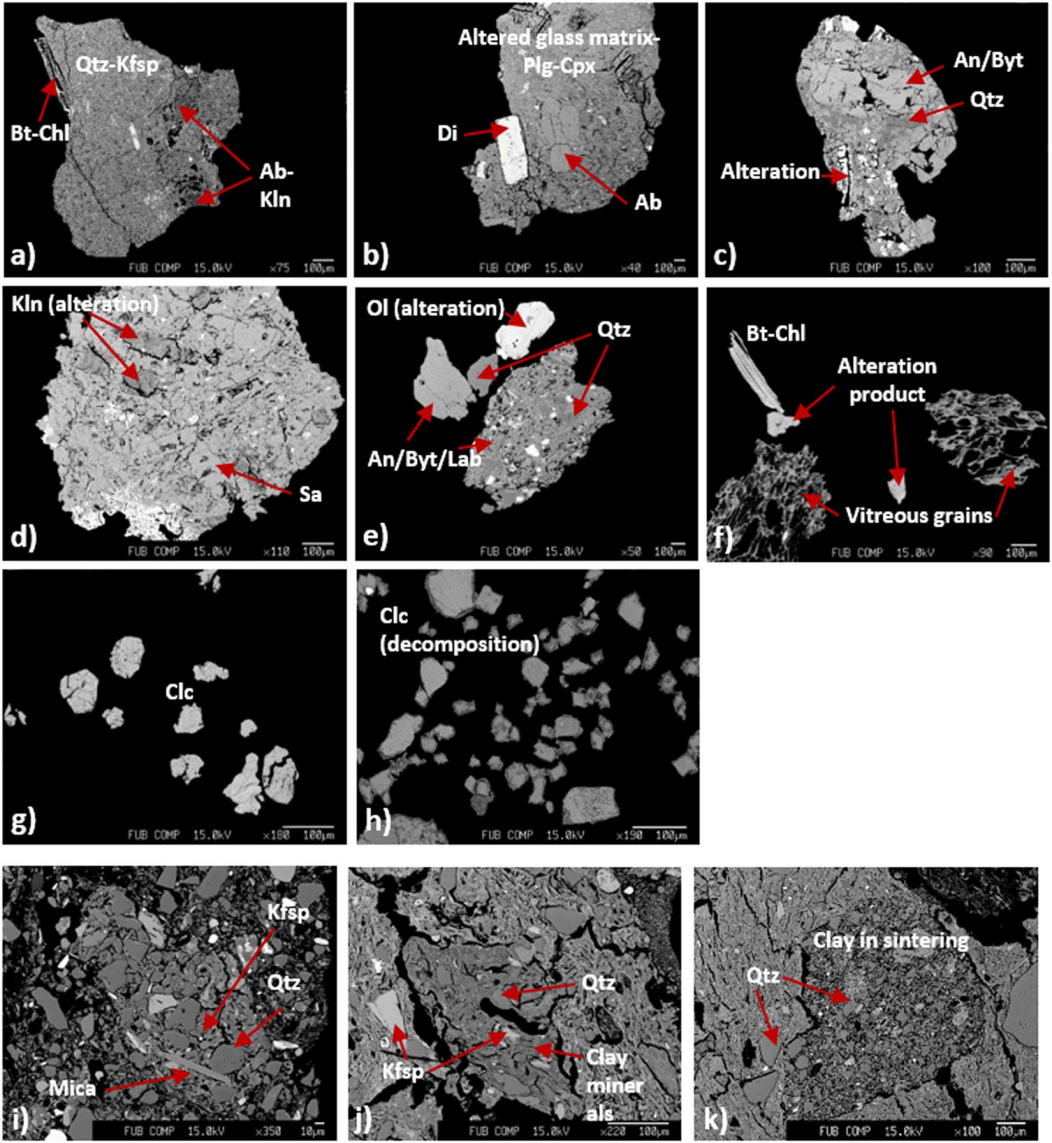
Mineralogical composition of sand grains in the ceramic paste (all scales for 100 µm): (**a**) quartz and K-feldspar matrix, biotite-chlorite and kaolinite-albite as alteration trace; (**b**) diopside and albite in altered glass matrix; (**c**) anorthite-bytownite, quartz and alteration product; (**d**) sanidine and kaolinite (alteration); (**e**) anorthite-bytownite-labradorite, quartz, olivine (alteration); (**f**) biotite-chlorite, alteration product, vitreous porous grains; (**g**) calcite; (**h**) calcite in thermal decomposition; (**i**) lithoclast composed of K-feldspar, quartz and mica; (**j**) aggregate composed of clay, K-feldspar and quartz; (**k**) aggregate composed of clay in sintering, quartz (Ab: albite, An: anorthite, Byt: bytownite, Bt: biotite, Chl: chlorite, Clc: calcite, Cpx: clinopyroxene, Di: diopside, Lab: labradorite, Ol: olivine, Plg: plagioclase, Qtz: quartz, Sa: sanidine).

Sand grains in the ceramics from Ransyrt 1 are categorized into four groups: (1) quartz and K-feldspar, mica-chlorite intergrowths with traces of albite and kaolinite; (2) quartz and K-feldspar, mica-chlorite intergrowths and plagioclase (from anorthite to albite, anhedral) and alteration products; (3) Plagioclase (albite, euhedral in altered volcanic glass) and clinopyroxene (diopside, euhedral/subhedral); (4) quartz, K-feldspar and calcite (Fig. 3a–c,g). In many samples, quartz and K-feldspar build a fine mixture in grains (Fig. 3a). Ceramics excavated at Kabardinka 2 contain different combinations: (1) quartz and K-feldspar (anhedral) often accompanied by kaolinizing phases; (2) quartz, K-feldspar, mica-chlorite intergrowths and plagioclase, mostly Ca-plagioclase from anorthite to labradorite in a subhedral or euhedral form located in the altered volcanic glass and kaolinizing phases; (3) quartz, K-feldspar, calcite; (4) quartz, K-feldspar, calcite and mica-chlorite intergrowth; (5) random combinations of quartz, K-feldspar, plagioclase (Ca-dominant, subhedral), mica-chlorite intergrowths, calcite, kaolinizing phases, alteration product similar to olivine or amphibole, clinopyroxene, and SiO_2_-rich porous and vitreous grains (Fig. 3d–f,g).

The Samples found at Levinsadovka-Saf’janovo have simpler combinations: (1) quartz dominant; (2) quartz and sediments composed of quartz, K-feldspar and various alteration phases (Fig. 3i). In those sediments, there are grains containing thermally transformed clays, quartz and K-feldspar (Fig. 3i–k). Several calcite grains were observed in all samples.

### Chemical compositions of the ceramic matrix (grains < 50 µm)

Chemical composition of the ceramic matrix provides the background for estimating the evolution of the ceramic matrix under heating. In order to avoid the grain size effect, only grains smaller than 50 µm were measured to characterize the ceramic matrix using SEM-WDS. The results were normalized to 100% concerning porosity and (crystal-)water content of the matrix (see the whole results in Supplementary Table S2).

**Figure 4:**
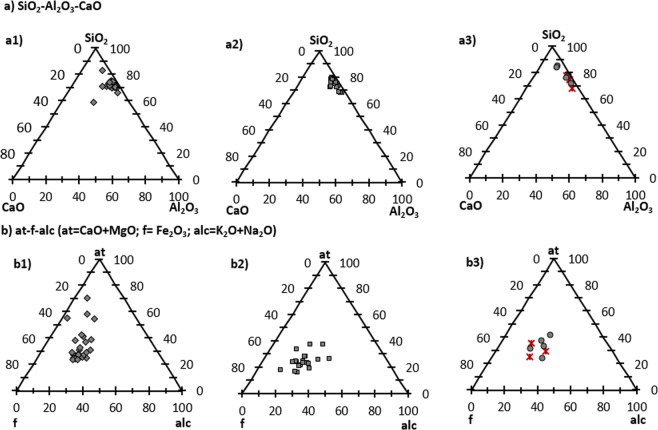
Chemical composition of ceramic matrix (grains < 50 µm) normalized to 100 wt.%: (**a**) SiO_2_-Al_2_O_3_-CaO: a1) Ransyrt 1; a2) Kabardinka 2; a3) Levinsadovka-Saf’janovo; (**b**) at-f-alc (at = CaO + MgO; f = Fe_2_O_3_; alc = K_2_O + Na_2_O): (b1) Ransyrt 1; (b2) Kabardinka 2, (b3) Levinsadovka-Saf’janovo.

The projection to the SiO_2_-CaO-Al_2_O_3_ system shows that samples from Levinsadovka and Saf’janovo have more SiO_2_ and those from Ransyrt 1 site the least (Fig. 4a). In the at-f-alc system (at = CaO + MgO; f = Fe_2_O_3_; alc = K_2_O + Na_2_O)^9^, Ransyrt 1 ceramics tend to have higher (Ca, −Mg) contents in the matrix, while those of Kabardinka 2 move toward Fe_2_O_3_ (Fig. 4b).

### Clay minerals in the ceramics

**Figure 5:**
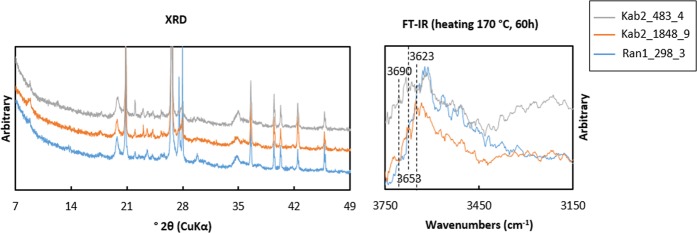
Representative XRD and FT-IR (transmittance, samples heated at 170 °C for 60 hours) results of ceramic sherds excavated at Ransyrt 1 (blue) and Kabardinka 2 (orange, gray).

Identified by the specific XRD Bragg peaks representing illite 1 M, such as set of 10 Å peak for (001), 4.98 Å for (002), 4.5 Å for (020) or 4.45–4.46 Å for (110), 2.58 Å for (130) or (13$\bar{1}$) lattice planes, illite is the main clay mineral of the studied ceramics (Fig. 5). Their higher FWHM distinguished illite from mica^10^. Samples without illite phase are either highly deformed by firing or they do not include any clay minerals. Illite could be confirmed by the transmittance IR vibrations at 3623–3630 and 3690 cm^−1^, too. The band occurring at 3623–3630 cm^−1^ is assigned to the stretching mode of the bond between Al and hydroxyl group which lies close to the SiO_4_ tetrahedral structure, ν(Al-OH)^11–13^. Some samples show a band at 3653 cm^−1^ related with ν(Al-OH) neighboring with AlO_4_ substitution of muscovite^14^. Although the peaks of quartz and feldspar present in all the ceramic samples overlap with those at ($\bar{1}\,$11) as well as ($\bar{1}\,$12)/(11$\bar{2}$) and (112) lattice plane of illite, (020)/(110) peaks according to the polytype lie between 4.5 and 4.45 Å regardless of the thermal transformation (see Supplementary Fig. S1 online)^15–18^. Therefore, the illite phase existed in ceramic pastes is supposed to be mixed layers of *cv*-1M and *tv*-1M polytypes.

### Firing behaviors of the illite based ceramics

**Figure 6:**
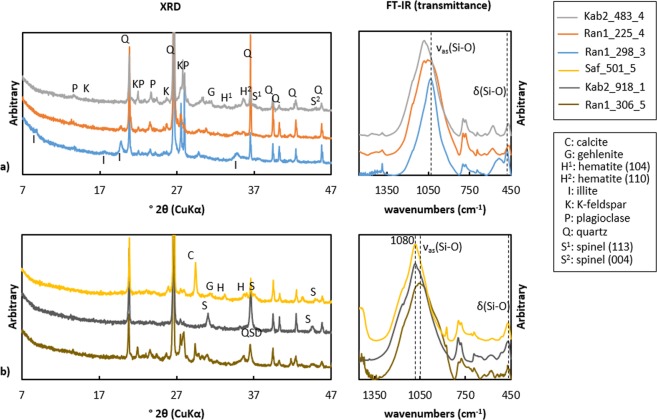
Example of comparison between XRD and FT-IR according to firing conditions: (**a**) three ceramic sherds from Ransyrt 1 fired in the oxidizing atmosphere with the estimated firing temperature of 300–675 °C (blue), 675–750 °C (orange), 1050–1300 °C (light grey); (**b**) three ceramic sherds fired at over 1050 °C (estimation) in Ca-rich matrix/reducing atmosphere (dark yellow), Ca-poor matrix/oxidizing atmosphere (dark grey); Ca-rich/oxidizing atmosphere (yellow).

The samples could be qualitatively ordered with respect to the decreasing illite XRD intensity that is taken as measure of increased thermal degradation (Fig. 6). While illite peaks are decreasing in intensity, new minerals are crystallizing in the ceramics. Once the peak at 2.58 Å of illite is dispersed, a new peak of hematite (110) lattice plane starts to grow. The main peak of natural hematite at (104) occurs later as a sub peak, as the illite peak decrease and the hematite peak for (110) gains more intensity. In highly modified ceramics by firing, the intensities of the both peaks for hematite become equal. Reddish color caused by hematite formation indicates oxidizing firing during ceramic production. Higher firing degree is confirmed by spinel peaks of (113) and (004). Dark gray or blackish-brown samples show decrease of illite peaks without hematite crystallization, so that it is assumed that they were fired in the reducing atmosphere. If the samples contain calcite, gehlenite is detected corresponding to the decreasing illite peaks. In some samples containing hematite or spinel, the XRD background is smoothly increased between 15 and 35° for 2θ° indicating a vitreous phase. Other minerals such as K-feldspar, anorthite or clinopyroxene were not counted as the indicator of the thermal transformation in ceramics, because of they already existed in the ceramic pastes in various sizes.

**Figure 7:**
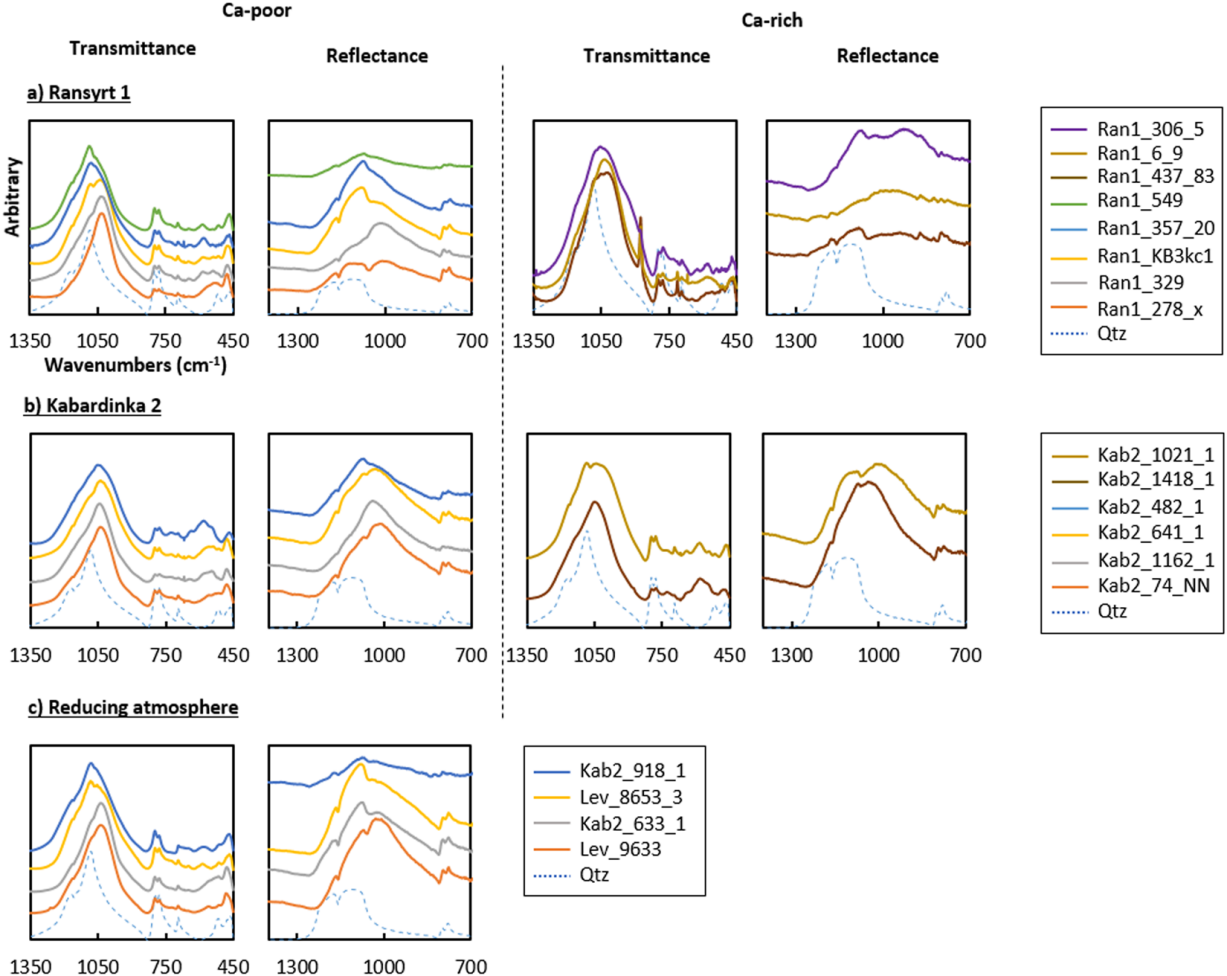
Comparison of FT-IR spectra in the transmittance mode (intensity normalized to 1) and reflectance mode (intensity from a 70 µm aperture size) of representative samples ordered according to the estimated firing degree: (**a**) Ransyrt 1 ceramics: Ca-poor matrix by transmittance and reflectance IR; Ca-rich matrix by transmittance and reflectance IR; (**b**) Kabardinka 2 ceramics: Ca-poor matrix by transmittance and reflectance IR; Ca-rich matrix by transmittance and reflectance IR; (**c**) ceramics fired in the controlled reducing atmosphere: Ca-poor matrix by transmittance and reflectance IR.

The main IR band in the transmission mode for the matrix ranges between 900 and 1080 cm^−1^, mainly affected by the asymmetric stretching mode of Si-O bonds in clays, ν_as_(Si-O). As proven by heating experiments^19–22^, samples with strong XRD peaks for illite have the main IR band between 1027–1030 cm^−1^, which is similar to the unfired illite, while samples with weaker illite peaks have this band shifted to the higher wavenumbers. Samples including hematite or spinel have often the main band at 1080 cm^−1^ assigned for quartz and sub band between 1050 and 1080. In some partly molten samples, only the main band at 1080 cm^−1^ appears without sub bands between 900 and 1100 cm^−1^, thereby indicating the total collapse of the illite structure. The changes of wavenumbers and spectral shapes of the main band visible between 1027/1030 and 1080 cm^−1^ coincide with the changes in wavenumbers assigned to the bending mode of Si-O-Si and O-Si-O bonds, δ(Si-O-Si) and δ(O-Si-O) from the higher to lower wavenumbers within 460–480 cm^−1^. This indicates that upon firing asymmetric deformation occurs in length and angle of Si-O bonds in the tetrahedral sheet. In Ca-rich ceramics containing gehlenite, the main FT-IR band shifts to the lower wavenumbers close to 920–930 cm^−1^ that is related to Ca-aluminosilicates^23^. The clay dominant area could be focused by reflectance IR using a 70 µm aperture size. It was performed on polished cross sections of the same samples and yields similar spectra to the transmittance IR in all cases (Fig. 7).

**Figure 8:**
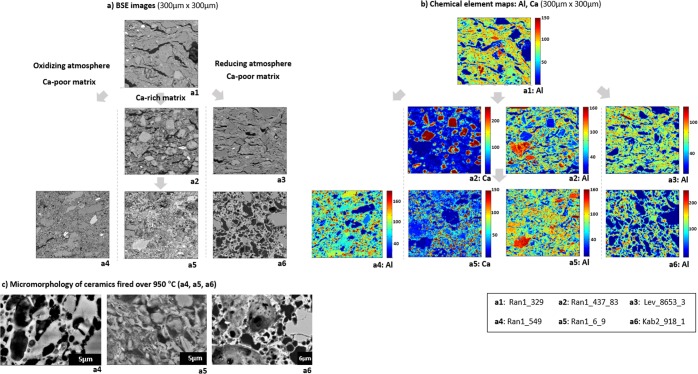
Comparison of general morphological changes of representative samples according to estimated firing temperature, Ca presence, and atmospheric conditions: (**a**) BSE images (300 µm × 300 µm); (**b**) Al distribution maps of together with Ca maps for Ca-rich matrix (300 µm × 300 µm); (**c**) comparison of micromorphology (BSE) between ceramic sherd fired over 950 °C (estimated).

Micromorphology of the ceramic matrix as visualized by BSE images and chemical element maps supports this relation between thermal transformation and crystallization observed by the XRD and FT-IR. This is summarized in Fig. 8, based on the parallelization of all three lines of evidence. If samples have strong illite XRD peaks and the main IR band close to 1027–1030 cm^−1^, their ceramic matrix features open pores with elongated shapes. If the samples have weak illite peaks and their main IR band occurs at higher wavenumbers, the fabric is characterized by globular and closed pores, often filled with aluminosilicate melts containing Mg, Fe and Ca as minor components, as SEM-EDS proved. The compositional ratio of (Fe + Mg + Ca)/Al or Si is ≤ 0.3 wt.%. In element distribution maps of Ca-poor samples containing hematite, Al is enriched in closed pores indicating the formation of Al-rich melt. In samples devoid of hematite but with their main IR band occurring at similar wavenumbers to that of hematite containing samples, the fabric shows already less pores between quartz or feldspar sand/silt grains and clay matrix, although the clay structures still exist in the samples. In the element distribution maps of these samples, there are less gaps between sand grains and clay matrix, in spite of still existing huge open pores. Samples containing spinel without hematite and illite show a matrix with closed pores, too. In Ca-rich ceramics, Ca-carbonate combines with aluminosilicate clays and gehlenite is formed in a globular or tabular shape at the surface of calcite grains. Spinel appears in all kinds of ceramics with highly progressed partial melting. Slight variations in d-spacings indicate various spinel solid composition. In this study, most calcite grains are partly decomposed forming a reaction rim at the grain boundary and interdiffusion of Ca^2+^ ions from original calcite grain into the porous ceramic matrix^24^ and Mg^2+^ ions from the clays into calcite occurs^25^ (see Supplementary Fig. S2). As a consequence, IR vibrations of (CO_3_)^2−^ groups, especially between 1430–1450 cm^−1^, move to slightly higher wavenumbers forming a broader band shape^26,27^. Table 1 summarizes the results from the micropore morphology analysis and XRD describing the presence and absence of indicator minerals, FT-IR (transmittance) of bulk ceramic powder mixed with KBr, in relation to the macroscopic colors of ceramics cross sections for representative samples.

**Table 1:** Description of cross section colors, micropore morphology, XRD peaks of pyrometamorphic minerals and main and sub bands of FT-IR (transmittance) vibrations for the representative samples (clc: calcite, geh: gehlenite, hem: hematite, ill: illite).

Sample No.	Ceramic color (cross section)	Micropore morphology	XRD (indicator minerals, peaks)	FT-IR (transmittance, Si-O stretching, cm^−1^)
Ran1_437_83	red brown (surface), dark brown	elongated open	ill(020)/(110), clc, geh trace	1024 (main), 1052&1080 (sub)
Ran1_225_4	dark brown, brown	elongated open, closed	ill (020)/(110) (130)/(13$\bar{1}$), hem (104) (110), clc	1054 (main), 1078 (sub)
Ran1_261_40	light brown	elongated open	ill (001) (002) (020)/(110) (130)/(13$\bar{1}$)	1035 (main)
Ran1_357_20	light/red brown	elongated open, closed globular	clc, geh, spl, hem (104) (110)	1079 (main), 1063 (sub)
Ran1_6_9	orange red	closed globular	ill (020)/(110), clc, hem (104) (110)	1034 (main)
Ran1_278_x	dark brown	elongated open	ill (001) (002) (020)/(110) (130)/(13$\bar{1}$)	1031 (main)
Ran1_554_4	brown (surface), dark brown	elongated open	ill (001) (002) (020)/(110) (130)/(13$\bar{1}$), hem (110) trace, clc	1028 (main)
Ran1_217_17	brown	elongated open	ill (001) (020)/(110) (130)/(13$\bar{1}$) hem (110) trace	1035 (main), 1080 (sub)
Ran1_244_9	red brown, dark brown	closed globular	hem (104) (110), clc	1035 (broad main between 1010 and 1050)
Ran1_326_9	light brown, gray brown	elongated open	ill (020)/(110) (130)/(13$\bar{1}$)	1034 (main), 1055 (sub)
Ran1_167_4	brown, gray brown, dark brown	elongated open, closed globular	ill (020)/(110) (130)/(13$\bar{1}$), hem (110) trace	1040 (main), 1050 (sub)
Ran1_509_9	gray brown	closed irregular	ill (020)/(110) (130)/(13$\bar{1}$), hem (110) trace	1034 (broad main between 1013 and 1053)
Ran1_17_2	light brown (surface), dark brown	elongated open	ill (001) (002) (020)/(110) (130)/(13$\bar{1}$), hem (110) trace	1034 (main)
Ran1_306_5	brown gray	closed globular	clc, geh, spl, hem (110)	1054 (main), 1076 (sub)
Ran1_549	orange red	closed globular	hem (104) (110)	1087 (main), 1062 (sub)
Ran1_470_ceramic	pink red	closed irregular	clc, hem (104) (110)	1080 (main), 1055 (sub)
Ran1_370_1	dark brown gray	elongated open, closed	hem (104) (110), clc, geh trace	1041 (broad main between 1030 and 1053)
Ran1_449	red brown, brown	elongated open	ill (001), (020)/(110), (130)/(13$\bar{1}$), hem (104) (110)	1041 (main), 1080 (sub)
Ran1_KB3kc1	red brown (surface), dark brown	elongated open	ill (020)/(110) (130)/(13$\bar{1}$), hem (110) trace	1039 (main), 1083 (sub)
Ran1_514_3	dark brown, brown	elongated open	ill (001) (020)/(110) (130)/(13$\bar{1}$), hem (110)	1039 (main), 1083 (sub)
Ran1_514_1	dark brown	elongated open	ill (020)/(110) (130)/(13$\bar{1}$), hem (110) trace	1035 (main), 1078 (sub)
Ran1_527_1	dark brown, brown	elongated open	ill (001) (020)/(110) (130)/(13$\bar{1}$), hem (110) trace	1034 (main), 1080 (sub)
Ran1_329	dark brown	elongated open	ill (001) (020)/(110) (130)/(13$\bar{1}$), hem (110) trace	1035 (main), 1074 (sub)
Ran1_514_2	dark brown	elongated open	none	1090 (main), 1040&1060 (sub)
Ran1_dmp1	red brown, dark brown	elongated open, closed globular	ill (001) (020)/(110) (130)/(13$\bar{1}$), hem (104) (110)	1053 (main), 1080 (sub)
KAE2007_2113_1	dark brown	elongated open	ill (001) (002) (020)/(110) (130)/(13$\bar{1}$), hem (110) trace	1085 (main), 1054 (sub)
KAE2008_844_1	light brown	elongated open	ill (001) (002) (020)/(110) (130)/(13$\bar{1}$), hem (110) trace	1084 (main), 1023 (broad sub 1005 and 1043)
KAE2008_633_1	black	elongated open	ill (020)/(110) (130)/(13$\bar{1}$), hem (110)	1039 (main)
KAE2007_1697_1	red brown (surface), dark brown	elongated open	ill (020)/(110) (130)/(13$\bar{1}$) trace	1084 (main), 1054 (sub)
KAE2007_1235_1	gray brown	elongated open	ill (001) (002) (020)/(110) (130)/(13$\bar{1}$) trace, clc	1039 (main)
KAE2007_1418_1	brown (surface), dark brown	elongated open, closed	ill (020)/(110) trace (130)/(13$\bar{1}$), hem (104) trace (110)	1049 (main)
KAE2007_NN	brown	elongated open	ill (001) (002) (020)/(110) (130)/(13$\bar{1}$)	1040 (main)
KAE2007_28/2	dark brown (surface), light brown	elongated open	ill (001) (002) (020)/(110) (130)/(13$\bar{1}$)	1038 (main)
KAE2007_797_1	red brown (surface), dark brown	elongated open	ill (020)/(110) (130)/(13$\bar{1}$) trace, clc	1041 (main), 1080 (sub)
KAE2007_918_1	dark gray	closed globular	spl	1084 (main), 1066 (sub)
KAE2007_482_1	dark red (surface), red	elongated open, closed globular	ill (130)/(13$\bar{1}$), hem (104) (110)	1053 (main)
KAE2008_1195_6	red brown (surface), dark brown	elongated open	ill (001) (002) (020)/(110) (130)/(13$\bar{1}$), hem (110) trace	1031 (main)
KAE2008_1162_1	black (surface), light brown	elongated open	ill (001) (002) (020)/(110) (130)/(13$\bar{1}$)	1030 (main)
KAE2008_516_2	red brown (surface), dark brown	elongated open	ill (001) (002) (020)/(110) (130)/(13$\bar{1}$), hem (104) (110)	1039 (main)
KAE2008_1021_1	light brown, gray brown	elongated open, closed	ill (020)/(110) trace	1084 (main), 1038 (broad sub between 1005 and 1055)
KAE2008_1152_1	light brown	elongated open	ill (001) (020)/(110) (130)/(13$\bar{1}$) trace	1020 (broad main between 1047–990), 1080 (sub)
KAE2008_516_26	light brown	elongated open	ill (001) (020)/(110) (130)/(13$\bar{1}$), hem (110)	1043 (main)
KAE2008_483_3	pink red (surface), dark gray	elongated open	ill (020)/(110) (130)/(13$\bar{1}$) trace	1080 (main), 1055 (sub)
KAE2008_641_1	light brown	elongated open	ill (020)/(110) (130)/(13$\bar{1}$), hem (104) trace (110)	1045 (main)
Lev_9633	Black, dark brown	elongated open	ill (001) (002) (020)/(110) (130)/(13$\bar{1}$)	1039 (main)
Lev_7718	light brown (surface), dark brown	elongated open	ill (001) (020)/(110) (130)/(13$\bar{1}$)	1042 (main)
Lev_8653_1	light brown, black	elongated open	ill (001) (020)/(110) (130)/(13$\bar{1}$), clc	1031 (main)
Lev_8653_3	black, dark gray	elongated open	ill (020)/(110) (130)/(13$\bar{1}$) trace, clc	1084 (main), 1054 (sub)
Lev_8653_4	light brown (surface), dark brown	elongated open	ill (020)/(110)	1084 (main), 1058 (sub)
Saf_501_5	orange red	closed globular	clc, geh, hem (104) (110), spl	1085 (main), 1078 (sub)
Saf_502_3	red brown (surface), dark brown	elongated open, closed	ill (020)/(110) trace (130)/(13$\bar{1}$) trace, hem (104) trace (110) trace, clc	1085 (main), 1060 (sub)

**Figure 9:**
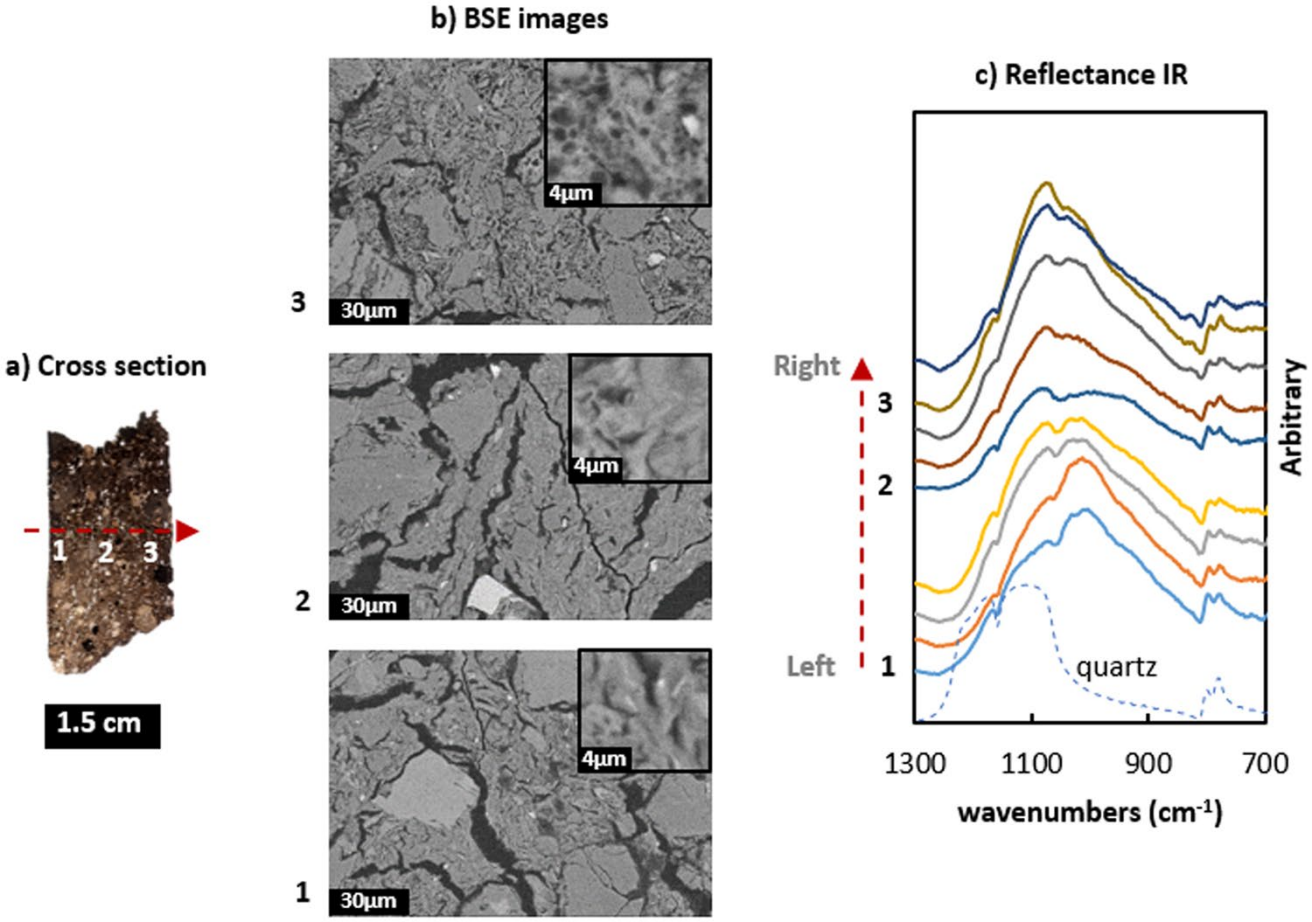
Changes in morphology and reflectance IR (aperture size: 70 × 70 µm^2^) within a single sherd (from left to right): (**a**) cross section of a ceramic sherd; (**b**) BSE images from left, middle and right part; (**c**) reflectance IR from left to right part on the cross section.

Figure 9 shows the reflectance IR profile across a single sherd with a 70 × 70 µm^2^ aperture size. Spatial distribution of different IR bands in terms of intensity, wavenumbers and shape was identified within a single sherd. The main band attributed to ν_as_(Si-O) varies from the left to the right side on the cross section. The band intensity around 1020 cm^−1^ decreases, while the intensity around 1080 cm^−1^ assigned to quartz increases. BSE images from areas along this profile depict a parallel switch from open to closed pores in the matrix. This method could distinguish the different ceramic part fired in the different atmospheric conditions. For example, a slip ware excavated at Kabardinka 2 had very similar mineralogical and chemical composition both for the body as well as the slip part (see Supplementary Table S1). Only the mechanical separation and the differences in the pattern of Fe distribution indicated a separate process for the preparation of the both parts (Supplementary Fig. S3c). Moreover, IR vibrations of ν_as_(Si-O) could prove that the body part was fired in the reducing atmosphere and the slip part in the oxidizing (Supplementary Fig. S3a,b).

In addition to the thermal transformation and crystallization in the ceramics, BSE images and reflectance IR of many samples detected an alteration layer at the surface of the samples, developed during the deposition after use. Almost all of those layers are under 100 µm thick from the surface of the ceramics.

### Estimation of average firing temperature

**Figure 10:**
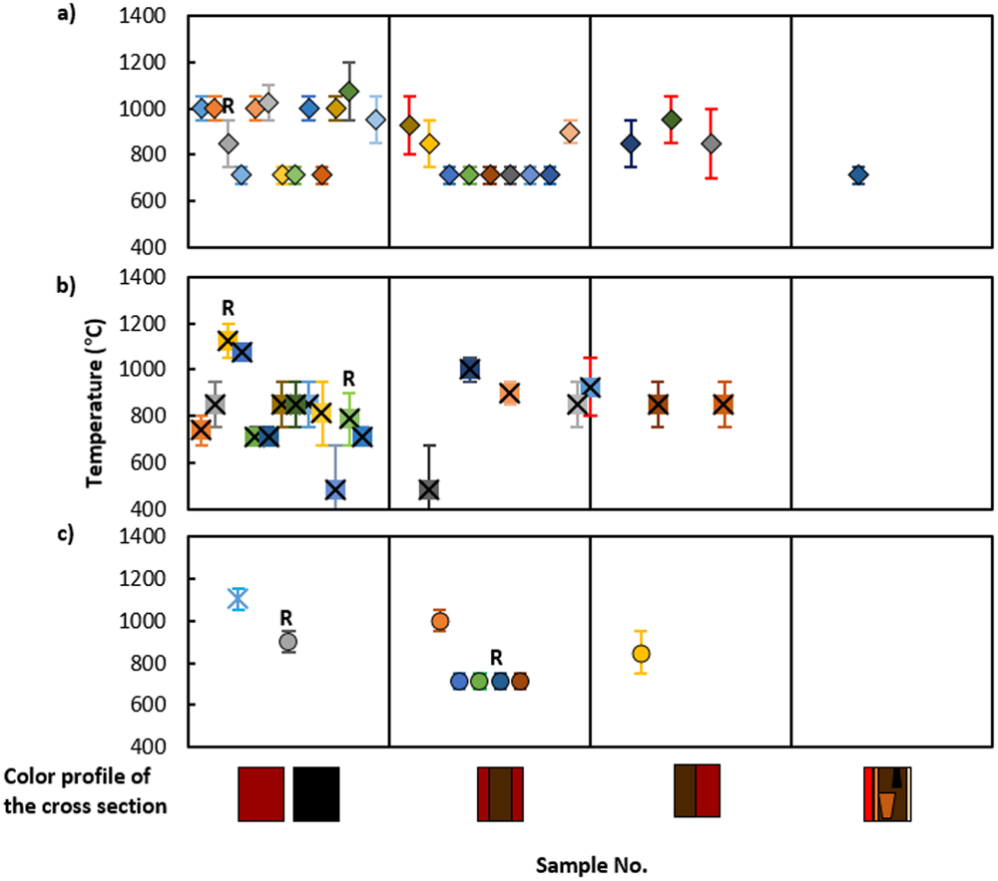
Average temperature of representative ceramic sherds according to the color profile of the cross section: (**a**) Ransyrt 1; (**b**) Kabardinka 2; (**c**) Levinsadovka-Saf’janovo (R: controlled reducing firing).

The dehydroxylation temperature for illite polytypes are 600 °C for *tv*-1M^28^, 750 °C for *cv*-1M^28^ and 525 °C for 2M_1_ Illite^29^. Other mineralogical transformations and crystallizations in the ceramics often accompany this thermal transformation of illite, too: transformation of goethite into hematite at 250–300 °C; thermal decomposition of Ca-carbonate at 650–700 °C; massive growth of hematite and spinel at 750 °C and 950–1050 °C; mullite crystallization at 1125 °C and its massive development at 1300 °C^30–32^. Additionally, morphological changes in a micrometer scale are clearly visible after the solid and liquid phase sintering of illite over 950 °C^33^. After solid state sintering, all grains are in contact to other grains and build a three dimensional network with interconnected pores of irregular shape in the matrix. At 1300 °C, it is degraded to a totally vitreous phase^34^. The changes in ν_as_(Si-O) and δ (Si-O) by FT-IR provided supplement data for the classification of the firing degrees. Figure 10 describes the distribution of average firing temperature depicted for each sample. The thicker red error bar for several samples indicates the spread of temperature detected within a cross section. In this estimation, ceramics from Kabardinka 2 were fired in more various temperatures in comparison to the other archaeological sites. Well controlled reducing firing could be detected at Kabardinka 2 and Levinsadovka. In the meanwhile, presence of calcite and its transformation to Ca-aluminosilicates as consequence in Ransyrt 1 ceramics, it is often difficult to confirm whether the potters pursued controlled reducing atmosphere. The ceramics from all the sites were not fully vitrified indicating the firing temperature below 1200–1300 °C.

## Discussion

Based on the serial employment of XRD, FT-IR (transmittance and reflectance) and SEM-EDS/WDS measurements of the illite based ceramics, firing conditions of number of heterogeneous archaeological samples could be reconstructed from the macro- to micro-scale. First, decreasing intensity and dispersed shape of XRD peak at 2.58 Å related with octahedral cations of all the polytypes give qualitative knowledge about the dehydroxylation in the samples during ceramic firing. Peaks at (001), (002), (020)/(110) seem to lose the intensity qualitatively corresponding to the thermal transformation, although these specific structures could still remain until the total collapse of the whole structure at 950 °C. In the samples, it was difficult to find out traces of illite 2M_1_ polytype related with the geothermal environment over 300 °C. The reason would lie in the fast firing process of the ceramics. Clay layers could not have enough time to stack with a regular rotating like the 2M_1_ type, due to the rapid changes during firing. This thermal transformation during ceramic firing process influences on IR vibrations corresponding to ν_as_(Si-O), δ(Si-O-Si) and δ(O-Si-O) in tetrahedral structure. This vibrational change becomes more visible after the beginning of illite dehydroxylation in the ceramic pastes. Water molecules derived from the reaction of two hydroxyl groups in the octahedral structure would cause clear deformation as they move out through the tetrahedral sheet and interlayer space or changes in from six to five Al-coordination in the octahedral structure influence of the structural deformation of the tetrahedral layer, too.

Before the total collapse of illite structure, the hematite peak at the (110) lattice plane appears in the decreasing illite XRD peaks as the result of oxidizing firing. Fe ions originated from illite would form hematite, because Mg and Fe substitute Al in octahedral sites or Fe replaces Si in tetrahedral sheet of illite^33,35^. According to the crystallization environment, the XRD Bragg peaks of hematite look different from those of hematite formed in the nature which has its strong main peak at (104) reflected by Fe^9^. During ceramic firing, the peak at (110) related with oxygen atoms of the crystal appears earlier at the lower temperature than the main peak at (104). It could indicate a deficiency of iron atoms in the earlier stage of crystallization, while oxygen atoms already occupied the hematite structure, similar to the phase transformation from goethite to hematite^36,37^. It can be also assumed that the crystal shapes of hematite in the earlier synthetic stage were nanorod characterized by the strong intensity at (110)^38^ which synthetic hematite shows as well^39,40^. This shape seems more likely to grow in the illite lath during illite dehydroxylation and solid state sintering stage. However, the lack of peak for (104) still indicates the Fe deficiency in the crystal and the lack of free ferric iron in the ceramic matrix. Maghemite peak at (311) which can also form through the dehydration and transformation of iron oxide/hydroxide, cannot be responsible for this peak, because no samples with less thermal deformation prove massive amounts of goethite, lepidocrocite or ferrihydrite in the ceramic paste, for which the dehydration of occurs earlier than the illite dehydroxylation. Those iron bearing minerals were observed only as individual aggregates often combined with anatase. The increasing degree of this peak doesn’t fit to the other peaks of anorthite, either. The peak for (104) lattice plane appears first after illite peaks are considerably low and the peak at the hematite (110) lattice plane is clearly visible. This peak grows further during the collapse and melting of the illite structure. This tendency can indicate the filling of Fe(III) position and crystal growth. In the melts, Fe does not remain as tetrahedrally-coordinated. Rather it fills octahedrally coordinated hematite, so that it can precipitate. Morphologically, the open elongated pores in the matrix gradually decrease and illite begins solid state sintering forming the clay lath. The closed pores become gradually surrounded and filled with the melts, after illite begins the liquid phase sintering between 900 and 950 °C in general^41^. These new pores have a globular shape due to interfacial tension effects^42^. Spinel would be crystallized before the total collapse of illite under 950 °C, as Mcconvill and Lee (2005) proved with their topotactical crystallization in the clay lath with TEM^43^. However, this mineral grows massively in the melt during the liquid phase sintering, as the XRD peaks at (113) and (004) proved. These peaks appear in the slightly different 2θ ° according to the samples indicating different unit cell parameters of this mineral in each ceramic samples. This thermally induced crystallization could occur similarly in the biotite-chlorite intergrowth as well^44^. In the Al-Si-Fe-Mg system of the melt, the chemistry for the nano-sized spinel crystals would be Si-Al spinel (2Al_2_O_3_·3SiO_2_), Mg-Al spinel (MgAl_2_O_4_) or Mg-Fe-Al spinel ((Mg,Fe)Al_2_O_4_). In comparison to the spinel crystallization, mullite, one of the common high firing mineral was not detected in any samples containing the liquid phase sintering, although this mineral can start to crystallize in the melt at 1100–1150 °C^45^. The absence of this mineral means that the peak firing temperature of the ceramics was under 1100–1150 °C, because the massive development of mullite occurs at 1300 °C. Alternatively, the firing time might be too short for enough Al released for the mullite formation. The very porous structure in a micrometer scale of the most samples in this study is related with that non-densifying mechanisms such as surface diffusion, lattice diffusion from the surface or vapor transport were stronger in this scale^46^. However, it is still unknown whether a densifying mechanism dominates the formation of illite laths in a nanometer scale, because shrinkage in the lath structure could cause porous fabrics for a micrometer scale.

In the Ca-rich ceramic paste, the pyrometamorphic process exhibits similarities to that of the Ca-poor matrix. At all the studied sites, Ca^2+^ ions were supplied from calcite, except of only one sample from Ransyrt 1 containing relatively high Mg contents which remind us of dolomite as the main bedrock building mineral of this site. According to the increasing firing temperature, hematite crystals grow and gehlenite is formed at the grain boundary of decomposing calcites. Because hematite can be developed in the high Fe_2_O_3tot_/CaO ratio, such as 0.7, the crystallization of hematite can prove the oxidizing firing of the Ca-rich ceramics^44^. In many cases, calcite and gehlenite, hematite and spinel (Mg-Al or Al-Si) coexist over 1050 °C, due to the heterogeneous mixed state of the ceramic pastes and huge grain size of calcite in the most ceramic pastes. If the local matrix in the paste has less Al and more Ca and Si, wollastonite is developed in a needle-like crystal shape, instead of gehlenite.

In the reducing atmosphere, lower *f*(O_2_), 1/T (absolute temperature) and Al/(Al + Si) stimulate the transformation of Fe into Fe(III)^47^. However, it is not crystallized as ferrous iron bearing minerals, but contributes to the earlier vitreous phases of the ceramic matrix cutting Si-O bonds like network-modifier in glass forming process^47–50^. Preexisting accessory phase composed of Fe/Ti oxides and hydroxides in the ceramic pastes, such as goethite would be transformed into magnetite, ilmenite or hercynite^51^, although these minor phases were not visible by XRD or FT-IR measuring the ceramic mixed powder.

**Figure 11:**
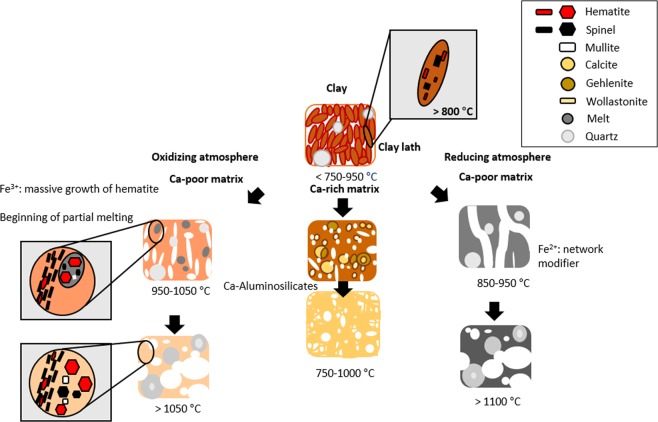
Morphological changes and new crystallization of ceramic composite materials under various firing conditions.

Concerning on the whole observations in the ceramic sherds, the pyrometamorphic process in the illite based ceramics between 300 and 1200/1300 °C is summarized in Fig. 11. Following reactions are describing representative phases during the firing process in the oxidizing atmosphere:

1. Ca-poor matrix in oxidizing atmosphere
  1 $$\begin{array}{l}\mathop{({\rm{K}},{{\rm{H}}}_{3}{\rm{O}}){({\rm{Al}},{\rm{Mg}},{\rm{Fe}})}_{2}{({\rm{Si}},{\rm{Al}})}_{4}{{\rm{O}}}_{10}[{({\rm{OH}})}_{2},({{\rm{H}}}_{2}{\rm{O}})]}\limits_{{\rm{illite}}}\to \\ \,\mathop{{{\rm{Fe}}}_{2}{{\rm{O}}}_{3}}\limits_{{\rm{hematite}}}+\mathop{({\rm{Mg}},{\rm{Fe}}){{\rm{Al}}}_{2}{{\rm{O}}}_{4}\,{\rm{or}}\,2{{\rm{Al}}}_{2}{{\rm{O}}}_{3}\,\cdot \,3{{\rm{SiO}}}_{2}}\limits_{{\rm{spinel}}}+\mathop{{{\rm{SiO}}}_{2}+\alpha }\limits_{{\rm{amorphous}}}\end{array}$$
2. Ca-rich matrix in oxidizing atmosphere

2 $$\begin{array}{l}\mathop{({\rm{K}},{{\rm{H}}}_{3}{\rm{O}}){({\rm{Al}},{\rm{Mg}},{\rm{Fe}})}_{2}{({\rm{Si}},{\rm{Al}})}_{4}{{\rm{O}}}_{10}[{({\rm{OH}})}_{2},({{\rm{H}}}_{2}{\rm{O}})]}\limits_{{\rm{illite}}}+\mathop{{{\rm{CaCO}}}_{3}}\limits_{{\rm{calcite}}}\to \\ \,\mathop{{{\rm{Ca}}}_{2}{\rm{Al}}({\rm{AlSi}}){{\rm{O}}}_{7}}\limits_{{\rm{gehlenite}}}+\mathop{{{\rm{Fe}}}_{2}{{\rm{O}}}_{3}}\limits_{{\rm{hematite}}}+\mathop{({\rm{Mg}},{\rm{Fe}}){{\rm{Al}}}_{2}{{\rm{O}}}_{4}\,{\rm{or}}\,2{{\rm{Al}}}_{2}{{\rm{O}}}_{3}\,\cdot \,3{{\rm{SiO}}}_{2}}\limits_{{\rm{spinel}}}+\mathop{{{\rm{SiO}}}_{2}+\alpha }\limits_{{\rm{amorphous}}}\end{array}$$

As minor phase, mullite in Ca-poor matrix and wollastonite in Ca-rich matrix could be present. In the reducing atmosphere, only spinel and amorphous silica were confirmed as main phase in the melts.

## Conclusion

The combination of various analytical methods enabled us to estimate firing conditions of the illite based ceramics in terms of firing temperature and atmosphere. Through the serial measurements covering from a lower to higher spatial resolution, a large number of heterogenous archaeological ceramics could be categorized into corresponding firing groups. Firing behavior of *cv*- and *tv*-1M illite and hematite, Ca-carbonate and spinel as well as chemical and mineralogical composition of the ceramic matrix composed of grains smaller than 50 µm delivered main information about the pyrometamorphic state of each sample. Additionally, relatively short firing time at the peak temperature would cause the various firing state even within a single sherd. Finally, the modelling suggested by this study describes possible firing behaviors of the number of illite based ceramics in detail. It will provide fundamental information about the firing place and installation/instrument, duration, placement of ceramics and atmospheric changes. Together with the archaeological contexts, it will contribute to reveal the regional development of technological styles in the daily ware production in the North Caucasus in the LBA and EIA.

## Methods

All ceramic sherds with the number of 70,000–80,000 excavated at Ransyrt 1 and Kabardinka 2 were optically investigated and categorized according to the surface color and color profile on the cross section, sand grains, textures and form. After this first evaluation, 138 ceramic samples representing various states of ceramic production techniques were selected for the detailed investigation. In order to compare to the mountain ceramics, 20 ceramic samples from Levinsadovka and Saf’janovo in the coast of Sea of Azov were investigated, too.

### Identification of ceramic pastes

The mineralogical composition of grains in various sizes within the ceramic pastes was investigated by various analytical methods such as polarized light microscopy, XRD, SEM-EDS/WDS. First, XRD patterns of the whole 158 samples were collected by the diffractometer, Empyrean by PANalytical in the measuring conditions of Cu Kα radiation (λ = 1.542 Å), 40 kV, 40 mA, 2θ range between 3–60 °, 0.013 ° for the 2θ step size and 50 s/step in the rotating mode. The samples were pulverized in a Tungsten mill for 4 minutes after the removal of the altered surface layer and measured without pre-treatment so that the specific peaks from heated clay minerals during ceramic firing can be distinguished from the possible regenerated or newly intruded clays in ceramics. Same samples were prepared for thin cross section with the thickness of 30–35 µm for the polarized light microscopy, too. According to the XRD and petrography results, polished thin sections of 52 representative samples coated with carbon were further investigated by Field emission scanning electron microscope by Zeiss SUPRATM 40 VP Ultra (thermal field emission type) with acceleration voltage 10–15 kV and Oxford Instruments EDX-System to identify minerals. BSE images were taken with an aperture size of 120 µm. For the supplement, SEM-EDS, JEOL JXA 8200 Superprobe with 15 kV acceleration voltage were employed, too.

In order to identify the dominant clay mineral in the original ceramic pastes, FT-IR transmittance measurements (Paragon 1000 PC by Perkin Elmer) were performed for supporting XRD data from above. 34 samples which contain less deformed structures were selected from 158 samples. 2–4 mg powder from each sample was mixed with KBr, pelletized and dried at 170 °C for 60 hours, in order to reduce the water amount adsorbed to pastes. After the dehydration, the samples were measured with 128 scans and 2 cm^−1^ spectral resolution between 450 and 4000 cm^−1^. Due to the higher noise ratio in 3000–4000 cm^−1^, it was necessary to apply the Savitzky-Golay filter with number of 13 points, in order to show the main clay phases clearly.

The chemical composition of the ceramic matrix was measured by SEM-WDS, JEOL JXA 8200 Superprobe using 5 crystal spectrometers for the major earth elements in an oxidized form and weight % (Na_2_O, BaO, FeO/Fe_2_O_3_, MgO, CaO, MnO, Al_2_O_3_, K_2_O, TiO_2_, SiO_2_, P_2_O_5_). The 52 polished thin sections from above were selected. Due to the porosity and (crystal-)water content in the ceramics, the total amount in weight % is normalized to 100.

### Identification of firing conditions

Three main analytical methods are employed for the estimation of the firing degree: XRD focusing on the specific peaks of clay minerals, FT-IR transmittance and reflectance measurements, micromorphological changes observed in BSE images from SEM.

FT-IR measurements were performed with Paragon 1000 PC by Perkin Elmer in transmission mode. For the sample preparation, powder from 116 ceramics selected from 158 samples were mixed with KBr and pelletized. The mid-IR curve is taken with 128 scans and a spectral resolution of 2 cm^−1^ for wavenumbers between 450 and 4000 cm^−1^. For this purpose, no smoothing filter was necessary. In order to focus more on the clay matrix within the heterogeneous mixed state of ceramic pastes, an IR-microscope (Bruker Hyperion 2000) attached to a Vertex 80 v FTIR-spectrometer and a MCT detector was employed to perform reflectance point analysis on the polished cross thin section of the 52 samples without carbon coating. Our main goal was the observation of Si-O stretching mode located between 900 and 1200 cm^−1^. The IR aperture size was fixed to 70 × 70 µm^2^, as this size was found to minimize the IR signal of the sand and silt grains and to maximize the signal of clay minerals. We performed 1024 scans with a spectral resolution of 2 cm^-1^. A silver mirror was used as reference for the IR reflectance experiments.

## Supplementary information

Supplementary information

## Data Availability

The datasets generated during and/or analysed during the current study are available from the corresponding author on reasonable request.
