# Supplementary material for: Pyrometamorphic process of ceramic composite materials in pottery production in the Bronze/Iron Age of the Northern Caucasus (Russia)
Source: Sci Rep. 2019 Jul 24;9:10725. doi: 10.1038/s41598-019-47228-y (PMC6656883; doi:10.1038/s41598-019-47228-y)
Supplement: Supplementary file 1 — Supplementary information [file 41598_2019_47228_MOESM1_ESM.docx]

Supplementary Information:

**Pyrometamorphic process of ceramic composite materials in pottery production in the Bronze/Iron Age of the Northern Caucasus (Russia)**

Ki Suk Park^1^, Ralf Milke^1^, Ilias Efthimiopoulos^2^, Regine-Ricarda Pausewein^3^, Sabine Reinhold^4^

^1^Institut für Geologische Wissenschaften, Freie Universität Berlin, Malteserstraße 74-100, 12249 Berlin

^2^Deutsches GeoForschungsZentrum GFZ, Section 3.6, Telegrafenberg, 14473 Potsdam

^3^RathgenForschungslabor, Staatliche Museen zu Berlin, Schloßstraße 1, 14059 Berlin

^4^Deutsches Archäologisches Institut, Im Dol 2-6, Haus II, 14195 Berlin

**Supplement 1. Chemical composition of altered grains and ceramic matrix**

According to the point analysis using SEM-WDS, alteration products in the ceramic pastes were identified as mica-chlorite mixed layers (Tab.S1: Ransyrt1_Bt_Chl_1-11; Kabardinka2_Bt_Chl_1). The mica-chlorite intergrowths in the Kabardinka 2 samples have more K contents than those from Ransyrt 1. The other phases (Tab.S1: Ransyrt1_alteration_1-6, Kabardinak2_alteration_1-10) are mostly dominated by SiO_2_, MgO, Fe_2_O_3_ and CaO. There are a few ceramic sherds containing diopside. In Kabardinka 2, the ceramics have vitreous and very porous Si rich grains (Tab.S1: Kabardinka2_X1-4). Numerous closed pores with a globular shape in a relatively big size could be produced by the fluid inclusions or during the melting phase.

| **Sample No.** | **SiO2** | **TiO2** | **Al2O3** | **FeO** | **MnO** | **MgO** | **CaO** | **Na2O** | **K2O** | **BaO** | **F** | **Cl** | **Total** |
| --- | --- | --- | --- | --- | --- | --- | --- | --- | --- | --- | --- | --- | --- |
| Ransyrt1_Bt_Chl_1 | 37.69 | 2.39 | 18.06 | 19.53 | 0.62 | 10.71 | 0.74 | 0.32 | 6.67 | 0.19 | 2.26 | 0.10 | **98.31** |
| Ransyrt1_Bt_Chl_2 | 40.67 | 3.02 | 20.62 | 14.35 | 0.41 | 9.15 | 1.11 | 0.40 | 6.41 | 0.16 | 1.78 | 0.15 | **97.43** |
| Ransyrt1_Bt_Chl_3 | 38.70 | 2.01 | 18.86 | 20.42 | 0.21 | 7.31 | 0.92 | 0.34 | 8.12 | 0.15 | 0.77 | 0.02 | **97.50** |
| Ransyrt1_Bt_Chl_4 | 36.03 | 0.17 | 23.04 | 28.30 | 0.73 | 6.86 | 1.78 | 0.13 | 1.23 | 0.07 | 0.00 | 0.05 | **98.36** |
| Ransyrt1_Bt_Chl_5 | 38.69 | 0.00 | 22.57 | 21.70 | 0.38 | 10.52 | 2.06 | 0.30 | 1.66 | 0.13 | 0.31 | 0.02 | **98.19** |
| Ransyrt1_Bt_Chl_6 | 36.96 | 0.35 | 21.59 | 24.84 | 0.39 | 12.29 | 2.30 | 0.17 | 0.72 | 0.06 | 0.21 | 0.00 | **99.80** |
| Ransyrt1_Bt_Chl_7 | 38.78 | 0.00 | 23.44 | 21.72 | 0.40 | 10.65 | 1.90 | 0.10 | 0.68 | 0.11 | 0.19 | 0.05 | **97.93** |
| Ransyrt1_Bt_Chl_8 | 38.91 | 0.00 | 19.25 | 24.08 | 0.24 | 11.55 | 2.70 | 0.09 | 0.30 | 0.05 | 0.34 | 0.01 | **97.37** |
| Ransyrt1_Bt_Chl_9 | 38.62 | 0.31 | 19.74 | 24.71 | 0.26 | 12.14 | 2.72 | 0.17 | 0.48 | 0.08 | 0.18 | 0.01 | **99.33** |
| Ransyrt1_Bt_Chl_10 | 37.73 | 10.01 | 15.44 | 15.27 | 0.15 | 6.12 | 13.45 | 0.05 | 0.33 | 0.22 | 1.50 | 0.02 | **99.67** |
| Ransyrt1_Bt_Chl_11 | 38.59 | 0.00 | 19.13 | 23.61 | 0.14 | 12.88 | 2.58 | 0.17 | 0.52 | 0.10 | 0.06 | 0.01 | **97.77** |
| Ransyrt1_alteration_1 | 51.03 | 0.54 | 3.90 | 5.67 | 0.06 | 15.82 | 23.32 | 0.16 | 0.03 | 0.00 | 0.08 | 0.00 | **100.58** |
| Ransyrt1_alteration_2 | 50.89 | 0.52 | 3.91 | 5.74 | 0.07 | 16.18 | 23.02 | 0.19 | 0.05 | 0.02 | 0.00 | 0.00 | **100.59** |
| Ransyrt1_alteration_3 | 50.00 | 0.57 | 4.64 | 6.88 | 0.08 | 15.34 | 22.10 | 0.22 | 0.00 | 0.04 | 0.25 | 0.00 | **100.00** |
| Ransyrt1_alteration_4 | 46.12 | 1.60 | 8.50 | 14.88 | 0.32 | 13.92 | 12.32 | 1.44 | 0.76 | 0.00 | 0.61 | 0.00 | **100.20** |
| Ransyrt1_alteration_5 | 46.27 | 1.70 | 8.20 | 13.51 | 0.26 | 13.41 | 12.32 | 1.53 | 0.77 | 0.00 | 0.65 | 0.00 | **98.33** |
| Ransyrt1_alteration_6 | 48.86 | 0.98 | 5.83 | 9.33 | 0.16 | 14.93 | 18.62 | 0.71 | 0.32 | 0.01 | 0.32 | 0.00 | **99.94** |
| Kabardinka2_Bt_Chl_1 | 39.44 | 4.51 | 13.06 | 19.05 | 0.17 | 13.59 | 2.21 | 0.26 | 4.76 | 0.05 | 0.65 | 0.09 | **97.55** |
| Kabardinka2_ alteration_1 | 45.15 | 1.48 | 8.08 | 18.38 | 0.26 | 12.21 | 12.38 | 1.33 | 0.82 | 0.03 | 0.30 | 0.00 | **100.30** |
| Kabardinka2_ alteration_2 | 44.41 | 1.97 | 9.16 | 17.73 | 0.36 | 10.83 | 12.47 | 1.55 | 0.95 | 0.00 | 0.64 | 0.00 | **99.78** |
| Kabardinka2_alteration_3 | 45.63 | 1.40 | 8.23 | 18.10 | 0.30 | 11.66 | 12.43 | 1.50 | 0.95 | 0.00 | 0.34 | 0.00 | **100.40** |
| Kabardinka2_alteration_4 | 44.53 | 1.97 | 9.36 | 17.74 | 0.30 | 10.93 | 12.21 | 1.57 | 0.81 | 0.06 | 0.34 | 0.00 | **99.67** |
| Kabardinka2_alteration_5 | 44.58 | 1.98 | 9.97 | 12.57 | 0.11 | 15.20 | 12.44 | 1.83 | 0.75 | 0.03 | 0.80 | 0.00 | **99.94** |
| Kabardinka2_alteration_6 | 45.67 | 1.95 | 9.40 | 12.44 | 0.15 | 15.60 | 12.01 | 1.67 | 0.66 | 0.04 | 1.36 | 0.00 | **100.36** |
| Kabardinka2_alteration_7 | 43.95 | 2.82 | 10.56 | 13.07 | 0.14 | 14.08 | 12.31 | 2.07 | 0.79 | 0.03 | 1.10 | 0.00 | **100.45** |
| Kabardinka2_alteration_8 | 38.25 | 0.14 | 24.07 | 12.73 | 0.19 | 0.08 | 24.35 | 0.00 | 0.12 | 0.00 | 0.08 | 0.04 | **100.00** |
| Kabardinka2_alteration_9 | 38.34 | 0.16 | 24.28 | 12.57 | 0.32 | 0.02 | 24.28 | 0.00 | 0.05 | 0.00 | 0.00 | 0.00 | **100.01** |
| Kabardinka2_alteration_10 | 38.24 | 0.11 | 24.28 | 12.68 | 0.25 | 0.00 | 23.91 | 0.01 | 0.07 | 0.02 | 0.04 | 0.04 | **99.64** |
| Kabardinka2_X_1 | 70.94 | 0.49 | 13.09 | 1.63 | 0.01 | 0.40 | 1.39 | 1.81 | 1.62 | 0.01 | 1.06 | 0.21 | **92.17** |
| Kabardinka2_X_2 | 73.00 | 0.16 | 13.68 | 1.69 | 0.03 | 0.43 | 1.54 | 2.16 | 2.84 | 0.05 | 0.16 | 0.00 | **95.65** |
| Kabardinka2_X_3 | 73.13 | 0.48 | 13.68 | 1.66 | 0.03 | 0.40 | 1.54 | 2.20 | 2.99 | 0.02 | 0.00 | 0.00 | **96.13** |
| Kabardinka2_X_4 | 74.47 | 0.22 | 13.78 | 1.80 | 0.01 | 0.41 | 1.35 | 2.16 | 3.49 | 0.07 | 0.00 | 0.00 | **97.75** |

**Table S1.** Quantitative analysis of mica-chlorite, alteration products and vitreous grains from Ransyrt 1 and Kabardinka 2

In order to figure out chemical environment of the ceramic matrix, major geological elements were measured by SEM-WDS and normalized to 100 wt% concerning on the porosity in the matrix (Tab.S2).

| **Ceramic No.** | **SiO2** | **TiO2** | **Al2O3** | **BaO** | **FeO** | **MnO** | **MgO** | **CaO** | **Na2O** | **K2O** | **P2O5** | **Total** |
| --- | --- | --- | --- | --- | --- | --- | --- | --- | --- | --- | --- | --- |
| Ran1_dmp1 | 61.70 | 0.62 | 22.64 | 0.11 | 6.52 | 0.15 | 1.91 | 1.66 | 1.10 | 3.25 | 0.34 | 100 |
| Ran1_549 | 60.34 | 0.77 | 23.85 | 0.13 | 7.38 | 0.08 | 1.87 | 2.07 | 0.49 | 2.50 | 0.52 | 100 |
| Ran1_329 | 62.30 | 0.61 | 21.41 | 0.14 | 6.82 | 0.13 | 1.61 | 2.02 | 0.99 | 2.89 | 1.06 | 100 |
| Ran1_527_1 | 61.05 | 1.00 | 18.77 | 0.07 | 8.21 | 0.14 | 1.69 | 3.84 | 1.08 | 2.91 | 1.24 | 100 |
| Ran1_514_2 | 59.65 | 0.72 | 22.25 | 0.05 | 7.95 | 0.15 | 1.67 | 2.87 | 1.32 | 2.55 | 0.83 | 100 |
| Ran1_370_1 | 59.42 | 1.01 | 17.51 | 0.06 | 5.40 | 0.06 | 5.02 | 6.49 | 0.66 | 3.52 | 0.84 | 100 |
| Ran1_514_3 | 64.52 | 0.75 | 18.86 | 0.06 | 6.66 | 0.10 | 1.64 | 3.18 | 0.78 | 2.42 | 1.02 | 100 |
| Ran1_449 | 59.90 | 0.75 | 19.34 | 0.05 | 6.86 | 0.09 | 2.43 | 5.14 | 0.63 | 4.40 | 0.41 | 100 |
| Ran1_514_1 | 63.46 | 0.75 | 18.23 | 0.06 | 6.44 | 0.11 | 2.05 | 3.88 | 0.88 | 2.84 | 1.29 | 100 |
| Ran1_KB3kc1 | 63.20 | 0.87 | 20.05 | 0.05 | 7.44 | 0.13 | 1.76 | 2.41 | 1.07 | 2.56 | 0.45 | 100 |
| Ran1_437_83 | 49.18 | 0.81 | 16.40 | 0.04 | 6.72 | 0.12 | 3.64 | 18.19 | 0.50 | 1.83 | 2.58 | 100 |
| Ran1_167_4 | 62.00 | 0.59 | 20.83 | 0.04 | 8.56 | 0.09 | 1.58 | 2.49 | 1.57 | 2.00 | 0.25 | 100 |
| Ran1_17_2 | 64.42 | 1.20 | 18.60 | 0.06 | 7.73 | 0.07 | 1.30 | 2.35 | 0.82 | 2.64 | 0.81 | 100 |
| Ran1_6_9 | 76.73 | 0.36 | 11.59 | 0.01 | 4.64 | 0.03 | 2.44 | 3.73 | 0.22 | 0.11 | 0.13 | 100 |
| Ran1_225_4 | 57.45 | 0.80 | 25.79 | 0.05 | 7.78 | 0.13 | 1.23 | 3.20 | 0.55 | 2.31 | 0.72 | 100 |
| Ran1_261_40 | 60.51 | 0.95 | 22.67 | 0.06 | 6.08 | 0.04 | 2.76 | 1.95 | 0.50 | 4.00 | 0.47 | 100 |
| Ran1_357_20 | 63.53 | 0.75 | 19.15 | 0.05 | 7.06 | 0.14 | 2.07 | 2.22 | 1.32 | 2.85 | 0.87 | 100 |
| Ran1_278_x | 58.11 | 0.83 | 20.58 | 0.06 | 8.38 | 0.15 | 2.86 | 4.30 | 0.38 | 2.69 | 1.64 | 100 |
| Ran1_217_17 | 62.00 | 1.03 | 22.86 | 0.06 | 5.54 | 0.13 | 0.96 | 2.81 | 0.86 | 2.64 | 1.12 | 100 |
| Ran1_244_9 | 62.63 | 0.89 | 18.12 | 0.05 | 6.95 | 0.27 | 2.95 | 4.43 | 1.04 | 2.48 | 0.20 | 100 |
| Ran1_326_9 | 61.73 | 0.77 | 19.54 | 0.07 | 6.55 | 0.29 | 2.34 | 4.82 | 0.69 | 2.36 | 0.84 | 100 |
| Ran1_306_5 | 59.68 | 0.75 | 15.53 | 0.05 | 6.54 | 0.09 | 5.07 | 8.74 | 0.98 | 2.23 | 0.34 | 100 |
| Ran1_111_1 | 60.31 | 0.92 | 22.49 | 0.04 | 8.36 | 0.10 | 1.09 | 2.63 | 1.17 | 2.29 | 0.58 | 100 |
| Ran1_224_9 | 64.03 | 0.83 | 19.82 | 0.07 | 7.35 | 0.19 | 1.46 | 1.94 | 0.88 | 2.76 | 0.67 | 100 |
| KAE_650_10 (body) | 67.78 | 0.74 | 16.44 | 0.05 | 8.07 | 0.21 | 1.65 | 1.36 | 0.64 | 2.96 | 0.10 | 100 |
| KAE_650_10 (slip) | 67.76 | 0.97 | 16.76 | 0.06 | 7.43 | 0.12 | 1.55 | 1.59 | 0.77 | 2.83 | 0.15 | 100 |
| KAE_1578_4 | 66.41 | 0.83 | 15.60 | 0.06 | 9.01 | 0.17 | 1.54 | 2.04 | 0.83 | 3.21 | 0.31 | 100 |
| KAE_2113_1 | 62.42 | 1.28 | 26.94 | 0.04 | 3.10 | 0.06 | 0.80 | 1.63 | 0.72 | 2.85 | 0.15 | 100 |
| KAE_844_1 | 64.07 | 0.84 | 19.78 | 0.04 | 8.72 | 0.03 | 1.10 | 1.29 | 1.07 | 2.67 | 0.38 | 100 |
| KAE_633_1 | 63.85 | 0.87 | 17.80 | 0.06 | 9.10 | 0.07 | 1.78 | 2.16 | 0.57 | 2.83 | 0.92 | 100 |
| KAE_2008_641_1 | 60.04 | 0.96 | 24.86 | 0.06 | 5.99 | 0.03 | 0.65 | 2.23 | 0.62 | 2.69 | 1.86 | 100 |
| KAE_483_3 | 66.58 | 1.03 | 16.25 | 0.06 | 8.42 | 0.18 | 1.46 | 1.73 | 0.78 | 2.87 | 0.65 | 100 |
| KAE_516_26 | 67.76 | 0.83 | 15.74 | 0.08 | 7.25 | 0.25 | 1.27 | 2.26 | 0.67 | 2.80 | 1.11 | 100 |
| KAE_516_2 | 66.98 | 0.72 | 15.94 | 0.07 | 7.12 | 0.05 | 1.30 | 2.07 | 1.13 | 3.04 | 1.57 | 100 |
| KAE_482_1 | 59.44 | 0.82 | 19.88 | 0.04 | 8.80 | 0.12 | 1.67 | 1.78 | 1.05 | 4.45 | 1.96 | 100 |
| KAE_918_1 | 53.80 | 0.95 | 22.60 | 0.05 | 14.63 | 0.26 | 1.83 | 2.05 | 0.43 | 2.70 | 0.71 | 100 |
| KAE_NN | 67.28 | 0.72 | 15.35 | 0.09 | 7.08 | 0.44 | 1.38 | 2.82 | 0.78 | 2.74 | 1.33 | 100 |
| KAE_1697_1 | 70.27 | 1.02 | 15.85 | 0.05 | 6.33 | 0.12 | 0.86 | 2.04 | 0.61 | 2.44 | 0.42 | 100 |
| KAE_797_1 | 59.74 | 0.80 | 16.95 | 0.05 | 10.91 | 0.33 | 1.62 | 5.69 | 0.89 | 2.66 | 0.38 | 100 |
| KAE_1162_1 | 65.53 | 1.15 | 22.26 | 0.05 | 4.43 | 0.02 | 1.05 | 1.77 | 0.74 | 2.77 | 0.23 | 100 |
| KAE_1195_6 | 61.08 | 0.98 | 20.46 | 0.07 | 8.91 | 0.38 | 1.44 | 2.61 | 0.89 | 2.35 | 0.82 | 100 |
| KAE_28_2 | 64.07 | 1.04 | 18.48 | 0.06 | 7.48 | 0.05 | 1.52 | 2.88 | 0.74 | 2.91 | 0.77 | 100 |
| KAE_1418_1 | 60.33 | 0.76 | 17.18 | 0.05 | 6.59 | 0.10 | 2.62 | 4.99 | 0.97 | 5.12 | 1.30 | 100 |
| KAE_1235_1 | 66.02 | 0.91 | 16.45 | 0.07 | 6.30 | 0.03 | 1.49 | 4.38 | 0.83 | 2.70 | 0.81 | 100 |
| KAE_1152_1 | 65.83 | 1.20 | 18.78 | 0.05 | 8.22 | 0.03 | 0.96 | 1.33 | 0.74 | 2.49 | 0.35 | 100 |
| KAE_1021_1 | 58.68 | 1.00 | 23.27 | 0.04 | 9.45 | 0.16 | 1.16 | 2.76 | 1.18 | 1.83 | 0.48 | 100 |
| Lev_7718 | 75.96 | 0.46 | 9.18 | 0.22 | 5.28 | 0.04 | 1.44 | 3.69 | 0.52 | 2.74 | 0.47 | 100 |
| Lev_8653_1 | 75.13 | 0.12 | 9.07 | 0.16 | 4.67 | 0.05 | 1.29 | 5.00 | 1.19 | 2.84 | 0.47 | 100 |
| Lev_8653_3 | 67.90 | 0.65 | 17.64 | 0.05 | 6.09 | 0.04 | 2.12 | 1.14 | 1.33 | 2.87 | 0.17 | 100 |
| Saf_501_5 | 58.83 | 0.89 | 24.64 | 0.03 | 7.01 | 0.04 | 1.99 | 3.40 | 0.31 | 2.62 | 0.23 | 100 |
| Saf_502_3 | 64.25 | 1.16 | 20.07 | 0.04 | 7.37 | 0.05 | 2.11 | 1.45 | 0.30 | 3.03 | 0.18 | 100 |
| Saf_501_4 | 67.70 | 1.04 | 17.31 | 0.06 | 5.29 | 0.04 | 1.56 | 2.25 | 0.47 | 3.54 | 0.73 | 100 |
| Lev_8653_5 | 64.41 | 1.03 | 22.16 | 0.04 | 5.88 | 0.02 | 1.62 | 2.24 | 1.10 | 1.32 | 0.19 | 100 |
| Lev_9633 | 65.91 | 0.77 | 16.90 | 0.06 | 6.34 | 0.06 | 2.25 | 3.08 | 1.17 | 3.16 | 0.31 | 100 |

**Table S2.** Chemical composition of the ceramic matrix (grains < 50 µm) normalized to 100 wt%

**Supplement 2. Changes in *cv*- and *tv*-1M illite mixed layers in ceramic pastes**

Illite d-spacing at the lattice plane (020) or (110) measured by XRD is very irregular, regardless of pyrometamorphic degrees of each samples (Fig.S1). Because *cv-*/*tv*-1M polytypes have different dehydroxylation temperatures and most illite phases in the nature occur in a mixed form, the illite phases in the ceramic pastes would be mixed layers [28].


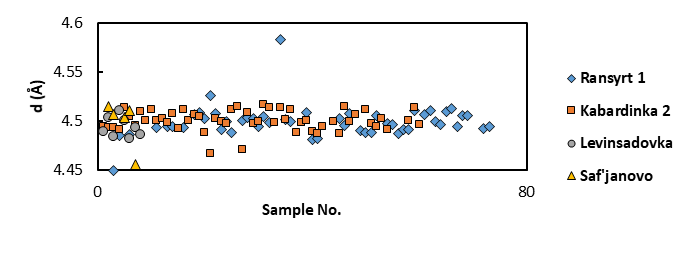


**XRD**

**Figure S1.** Illite d spacing at the lattice plane (020)/(110)

**Supplement 3. Calcite thermal decomposition in the ceramic pastes**

If amounts of Mg elements increase in the Ca-carbonate building dolomite, magnesite and aragonite, the absorbance IR peak at 1430 cm^-1^ assigned for the asymmetric stretching band of CO_3_^-2^ in calcite shifts to the higher wavenumbers [27].


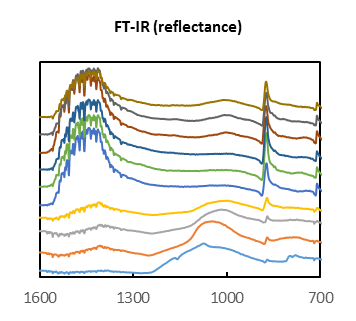


**b)**

**wavenumbers (cm^-1^)**

**Arbitrary**

**c)**


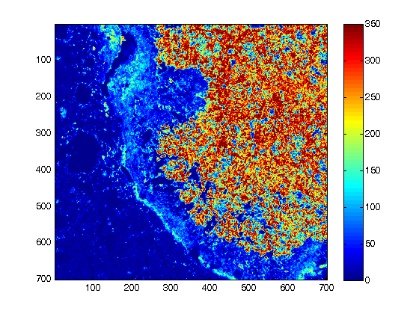


**1**

**10**

**8**

**100**

**200**

**300**


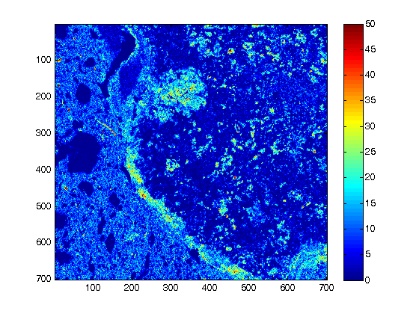


**1**

**10**

**8**

**20**

**40**


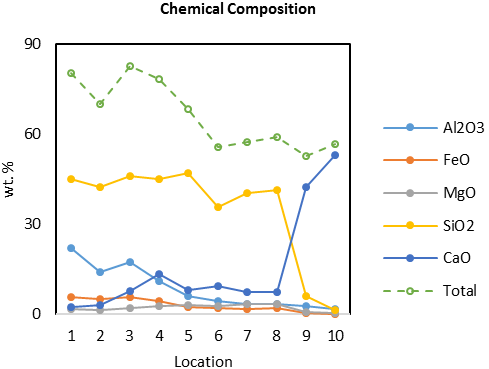

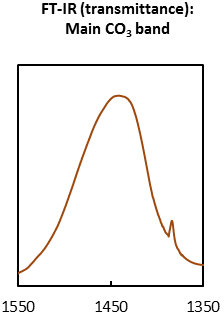


**1445 cm^-1^**

**Unheated Calcite: 1430 cm^-1^**

**a)**

**wavenumbers (cm^-1^)**

**Arbitrary**

1

8

9

10

**Figure S2.** Thermal decomposition of calcite: a) FT-IR (transmittance) of the ceramic paste containing decomposed calcite grains; b) FT-IR (reflectance) from different positions from the boundary through the whole calcite grain (c); c) Ca, Mg distribution maps (700 µm x700 µm) and chemical compositional changes according to different positions from the boundary to the calcite core measured by SEM-WDS (beam size 10 µm, without CO_2_ composition)

In case of calcite grains during firing over 650-700 °C in the ceramic paste, similar vibrational changes were observed (Fig.S2a). In the reaction rim, reflectance IR intensity of Ca-carbonate vibrations decreases, while the possible ν_as_(Si-O) gains the intensity (Fig.S2b). Shoval et al. (2011) reported similar phenomena in ceramic pottery measured with FT-IR (transmittance) and found the reason in Mg incorporation from clays into calcite. Ca and Mg distribution maps show the interdiffusion between these two elements through the reaction rim (Fig.S2c) [25]. The chemical composition of calcite grains and grain boundaries was measured by SEM-WDS with a beam size 10 µm, in comparison to the total weight % of each points without contribution of CO_2_.

**Supplement 4. Slip ware with decoration from the two step firing process**

**Fe distribution**


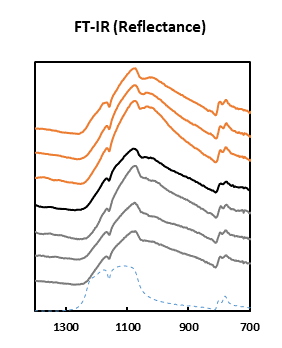


**1**

**4**

**2**

**3**

**5**

**6**

**7**

**Qtz**

**Body**

**Slip**

**Intermediate**

**Right**

**Left**

**b)**


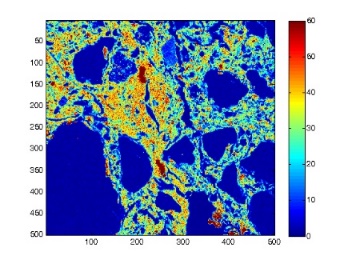

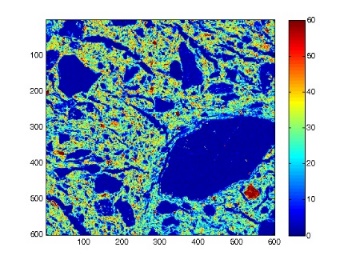

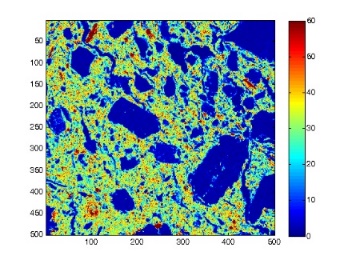


**c-1**

**c-2**

**c-3**


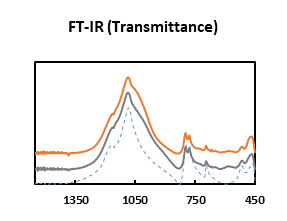

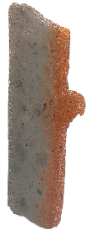


**Body**

**Slip**

**1**

**4**

**7**

**1 cm**


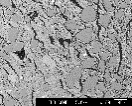

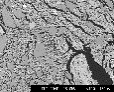

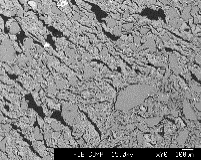

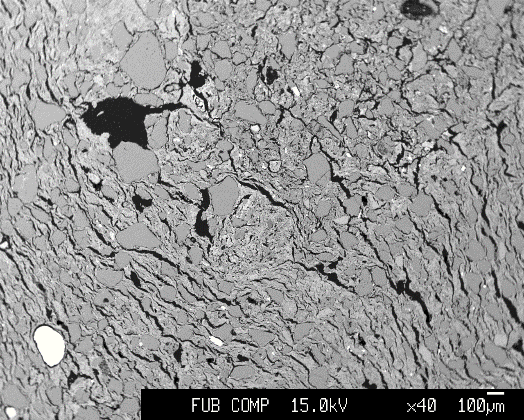


**c-1**

**c-2**

**c-3**

**1 mm**

**Slip**

**Body**

**Body**

**Slip**

**Qtz**

**a)**

**c)**

**Figure S3.** Cross section of a slip ware: a) FT-IR (transmittance) for the body and slip part; b) FT-IR (reflectance) across the cross section; c) BSE images between the slip and body part and Fe element maps (c-1: 500µm x 500µm, c-2: 600µm x 600µm, c-3: 500µm x 500µm)

In Kabardinka 2 ceramics, one sherd is assumed as a slip ware composed of a red-pink slip and grey body, which was produced probably from two step firing process: 1) reducing atmosphere, 2) oxidizing atmosphere (Fig.S3a). This slip ware has a relatively clear structural difference between the slip and the body, so that the different firing atmospheres by the potters could be clearly distinguished (Fig.S3b). The both parts have very similar mineralogical and chemical compositions, as described above, although Fe element maps acquired from the intermediate phase show clear distinction (Fig.S3c:1-3). Absorbance and reflectance IR spectroscopy revealed different firing degrees for the slip and body part. This result supports that the slip part was fired at the lower temperature than the ceramic body.
